# Monoclonal antibodies targeting the synthetic peptide corresponding to the polybasic cleavage site on H5N1 influenza hemagglutinin

**DOI:** 10.1186/1423-0127-19-37

**Published:** 2012-04-03

**Authors:** Henry J Tsai, Li-Ann Chi, Alice L Yu

**Affiliations:** 1 Genomic Research Center, Academia Sinica, Taipei, Taiwan; 2 Development Center for Biotechnology, Hsi-Chih City, Taiwan; 3 Department of Health and Nutrition, Asia University, Taichung, Taiwan

**Keywords:** Monoclonal antibody, Polybasic cleavage site, Hemagglutinin, H5N1 influenza

## Abstract

**Background:**

Avian influenza H5N1 virus is highly pathogenic partially because its H5 hemagglutinin contains a polybasic cleavage site that can be processed by proteases in multiple organs.

**Methods:**

Monoclonal antibodies (mAb) specific to the synthetic peptide of hemagglutinin polybasic cleavage site of H5N1 virus were raised and tested for their neutralizing potential.

**Results:**

Purified mAb showed suppression of H5N1 pseudovirus infection on Madin-Darby Canine Kidney (MDCK) cells but the efficacy was less than 50%. Since those mAb are specific to the intact uncut polybasic cleavage site of hemagglutinin, their efficacy depends on the extent of hemagglutinin cleavage on the viral surface.

**Conclusions:**

Proteolytic analysis suggests the low efficacy associated with those mAb may be due to proteolytic cleavage already present on the majority of hemagglutinin prior to the infection of virus.

## Background

Avian H5N1 highly pathogenic influenza virus was first isolated from sick geese in China during 1996 and later transmitted to human in Hon Kong during 1997 [1]. This H5N1 virus was spread throughout Asia and over as far as Europe or Africa by migratory birds in 2005 [1], which prompted a fear of global pandemic. Avian H5N1 Influenza virus has two major antigenic surface proteins, hemagglutinin (HA) and neuraminidase (NA), and a RNA genome which accumulates mutations rapidly over its life cycles [2]. The rapid accumulation of genomic mutations results in frequent alterations on the surface epitopes that is known as antigenic drift [3]. The function of HA is to recognize host sialic acid residue as an entry receptor [4,5], and to fuse viral envelope with vesicle's membrane [5,6] after the linker peptide between subdomain HA1 and HA2 of HA is cleaved by host trypsin-like proteases. Virulent H5 and H7 hemagglutinins [7] have a polybasic cleavage site that is exposed and cleavable by furin or other proprotein convertases [8,9] which enables the virus to infect multiple organs and leads to multisystem failure [7]. A second factor correlating to the high pathogenicity of H5N1 influenza virus is the PB2 subunit in polymerase complex [10,11]. The adaptation of viral polymerase complex to replicate in mammalian host cell is an important factor for the high pathogenicity associated with influenza virus [12,13]. The combination of polybasic H5 HA and humanized PB2 in the avian H5N1 virus makes it highly pathogenic and a pandemic possible with high mortality and morbidity similar to that of 1918, if this H5N1 virus ever adapts to human cell's entry receptor with an α-2,6 sialo-galactose linkage [14].

There are many antiinfluenza measures available. For example, vaccination is a good defense against highly pathogenic influenza like the avian H5N1 virus [15,16], but antigenic drift associated with influenza virus enables its evasion from host immunity and necessitates vaccination every year/season. Amantadine and Rimantadine target viral M2 channel protein during the viral endocytosis [17], but amantadine suffers from the prevalence of drug resistant viruses [18] and both compounds possess side effect on host central nervous system [19]. Oseltamivir and Zanamivir target viral neuraminidase activity during viral budding [17], but avian H5N1 as well as seasonal influenza viruses resistant to Oseltamivir have been reported [20-22]. Ribavirin targets viral polymerase activity, but its side effect is a major concern [23]; therefore, a new approach of suppressing influenza virus infection is highly desirable.

An antibody targeting the conserved epitopes on viral surface may be able to circumvent the antigenic drift and thus avoid the hit-and-miss situation associated with influenza vaccines. For example, the ectodomain of M2 channel protein is highly conserved among most strains of influenza A viruses and has been targeted as a broad spectrum epitope, but the antibody will only work on influenza A viruses and as the mutations accumulate at the ectodomain of M2 protein, they count against the efficacy of such antibody [24]. The HA2 domain of hemagglutinin is also conserved but is much more hydrophobic when compared to HA1 domain [25], possibly due to its role at facilitating membrane fusion during viral infection [5,6]. Few antibodies specific to this hydrophobic region have been reported so far [25] but antibodies of this type enjoy a broad spectrum reactivity [26-29]. For example, a pan influenza A antibody specific to an HA2 epitope was able to recognize all 16 subtypes of HA and neutralize group1 H1 and group 2 H3 [27].

The polybasic cleavage site on hemagglutinin is highly conserved among those highly pathogenic H5N1 viruses and its polybasic residue constituent should make this peptide fairly antigenic but discernable from other hydrophobic peptides and, therefore, this polybasic peptide is an interesting candidate as a broad spectrum epitope. Because the proteolytic cleavage of HA is a necessary step for an influenza virus to become infectious, we hypothesize that monoclonal antibody (mAb) specific to the polybasic cleavage site on hemagglutinin may be able to suppress virus infection by preventing HA cleavage by host proteases.

## Methods

### Antigenic epitopes

The polybasic cleavage site on hemagglutinin is highly conserved among H5N1 avian influenza viruses [7,30,31], but to ensure a successful raise of hybridoma cell lines, two different constructs were derived from the polybasic cleavage site, RERRRKKR, when raising mAb. One construct, RERRRKKR↓GLFGAIAGFI-ovalbumin (OVA, ↓ depicts HA cleavage site) gave rise to 3 monoclonal hybridomas, clone 3C4, 4H2, and 6B8; whereas RERRRKKR↓GLFGAIAC-keyhole limpet hemocyanin (KLH) gave rise to clone A, B, C, and D. Each mouse was immunized with approximately 50 μg antigen which was emulsified in 150 μL of Freund's complete adjuvant and boosted with the same dose of antigen but emulsified in incomplete adjuvant 14 days later.

### Purification of mAb

Those 7 hybridomas were cultured to raise ascitic fluid in nude mice and the mAb was purified by protein A resin (Pierce/Thermo Scientific, Rockford, IL). Briefly, monoclonal antibodies in the ascitic fluid was precipitated by 50% saturation of ammonium sulfate and redissolved in a minimal volume of phosphate buffered saline (PBS) before an overnight dialysis. Dialysed ascitic fluid was loaded onto a protein A column that had been equilibrated with 20 mM sodium phosphate pH7 buffer. The monoclonal antibody was eluted with 100 mM acetic acid pH2.8 and neutralized by 1 M Tris/HCl pH9. The immunoglobulin in elution was concentrated with Centricon (Millipore, Billerica, MA) and the contents were determined by Bradford protein assay (BioRad, Hercules, CA).

### Pepsin treatment of mAb

One mg of pepsin (Sigma P6887) was predissolved in 200 μL of 0.1 M glycine acetate (pH 2.6). Twenty μL of mAb was added with an equal volume of pepsin and digested at 37°C for 40 min before 1 μL of 1 M Tris (pH 9) was added to alleviate the protein precipitation due to acidity. The mixture was further incubated for 30 min before 4 μL of 1 M Tris (pH9) was added to stop the reaction. An aliquot of 225 μL D-MEM was added to the antibody/pepsin mixture and 50 μL of the mixture was used for pseudovirus neutralization assay.

### Epitope peptide analysis

Three synthetic peptides were used to evaluate the epitope binding property of those 7 purified monoclonal antibodies. The full length 18 meric peptide, RERRRKKRGLFGAIAGFI, the N-half 8 meric peptide, RERRRKKR, and the C-half 10 meric peptide, GLFGAIAGFI, were synthesized at the Genomic Research Center of Academia Sinica. All 3 peptides were dissolved in DMSO at 2 mg/mL concentration. An aliquot of 5 μL (10 μg) was spotted on a 1 cm square of PVDF membrane (Perkin-Elmer, Waltham, MA). Four ten-fold serial dilutions were also spotted on the membrane similarly. The PVDF membranes were allowed for air dry and subject to immunoblotting after the membrane being rewetted by methanol/PBS. Blocking of the PVDF membrane was carried out with 2% non-fat milk dissolved in PBS. The purified monoclonal antibodies were diluted 500 fold in 2% non-fat milk and allowed for 1 hr incubation with the membrane. Horseradish peroxidase (HRP) conjugated anti-mouse 2^nd ^antibody was used to probe the membrane at 1:5000 dilution for 1 hr. The immunoreactive spots were developed with a chemiluminescent substrate (Invitrogen, Carlsbad, CA) and scanned with a typhoon scanner (Amersham).

### Pseudovirus

To avoid using those highly pathogenic H5N1 viruses, pseudoviruses comprised of HA5 and NA1 antigens, Pol and Gag of HIV and a firefly luciferase reporter gene [32] were kindly provided by Min-Wei Chen with permission from Dr. David Ho. Three different H5N1 strains of pseudoviruses were used in the neutralization assay: 1) Vietnam 1194 H5N1 pseudovirus that has the consensus sequence, RERRRKKRGLFGAIAGFIEGG; 2) Turkey 2005 H5N1 pseudovirus that has an R339G mutation in its sequence, GERRRKKRGLFGAIAGFIEGG, mutation underlined; 3) Anhui 2005 H5N1 pseudovirus, RERRR_KRGLFGAIAGFIEGG, which has a deletion of K344 or K345 at the polybasic site. The peptide sequence of antigens and viral cleavage sites were listed in Table 1 for comparison. A vesicular stomatitis pseudo-virus (VSV) was employed as a negative control. All pseudoviruses were reconstituted in 293 T human embryonic kidney cells that were cultured in D-MEM medium (Gibco/Invitrogen, Carlsbad, CA) with 10% fetal bovine serum (FBS) supplement (Gibco/Invitrogen).

**Table 1:** Peptide sequence alignment of antigen constructs and hemagglutinin cleavage sites of Vietnam 1194, Turkey 2005, and Anhui 2005 viruses

Construct-1	RERRRKKR↓GLFGAIAGFI-***OVA***
Construct-2	RERRRKKR↓GLFGAIAC-***KLH***
Vietnam 1194	RERRRKKR↓GLFGAIAGFIEGG
Turkey 2005	GERRRKKR↓GLFGAIAGFIEGG
Anhui 2005	RERRR K R↓GLFGAIAGFIEGG

↓ depicts protease cleavage site.

*OVA*: ovalbumin; *KLH*: keyhole limpet hemocyanin

### Pseudovirus infection assay

Pseudovirus infection assay was performed on Madin-Darby Canine Kidney (MDCK) cells which were cultured in D-MEM supplemented with 10% FBS. Overnight cultured MDCK cells were incubated with pseudovirus in the presence or absence of mAb for 1 or 4 hours at 37°C before the pseudovirus mixture was removed by aspiration. Pseudovirus treated MDCK cells were cultured 48 hours further before the infectivity was determined. The infectivity of pseudovirus was determined by assaying the transfected and expressed luciferase activity in those MDCK cells. The MDCK cells were rinsed with PBS before being solublized with 60 μL Glo Lysis Buffer of Promega (Madison, WI) for 2 hours. Fifty μL of cell lysate was mixed with an equal volume of Bright-Glo Luciferase Substrate in an opaque well/plate. The chemiluminescence was detected with a TopCount NXT of Perkin Elmer (Waltham, MA).

### Immunoblot and densitometry analysis

The immunostain blot was carried out with a 4-20% gradient SDS-polyacrylamide gel and transferred to a PVDF membrane in a semidry blot transfer apparatus (BioRad). Both the primary and the secondary antibodies were diluted 1:5000 fold in PBS containing 2% non-fat milk. The stains were developed with chemiluminescent substrates of Invitrogen (Carlsbad, CA) and the band intensities were measured by a Typhoon scanner and analyzed by ImageQuant TL of GE Healthcare (Piscataway, NJ).

## Results and discussion

**Figure 1:**
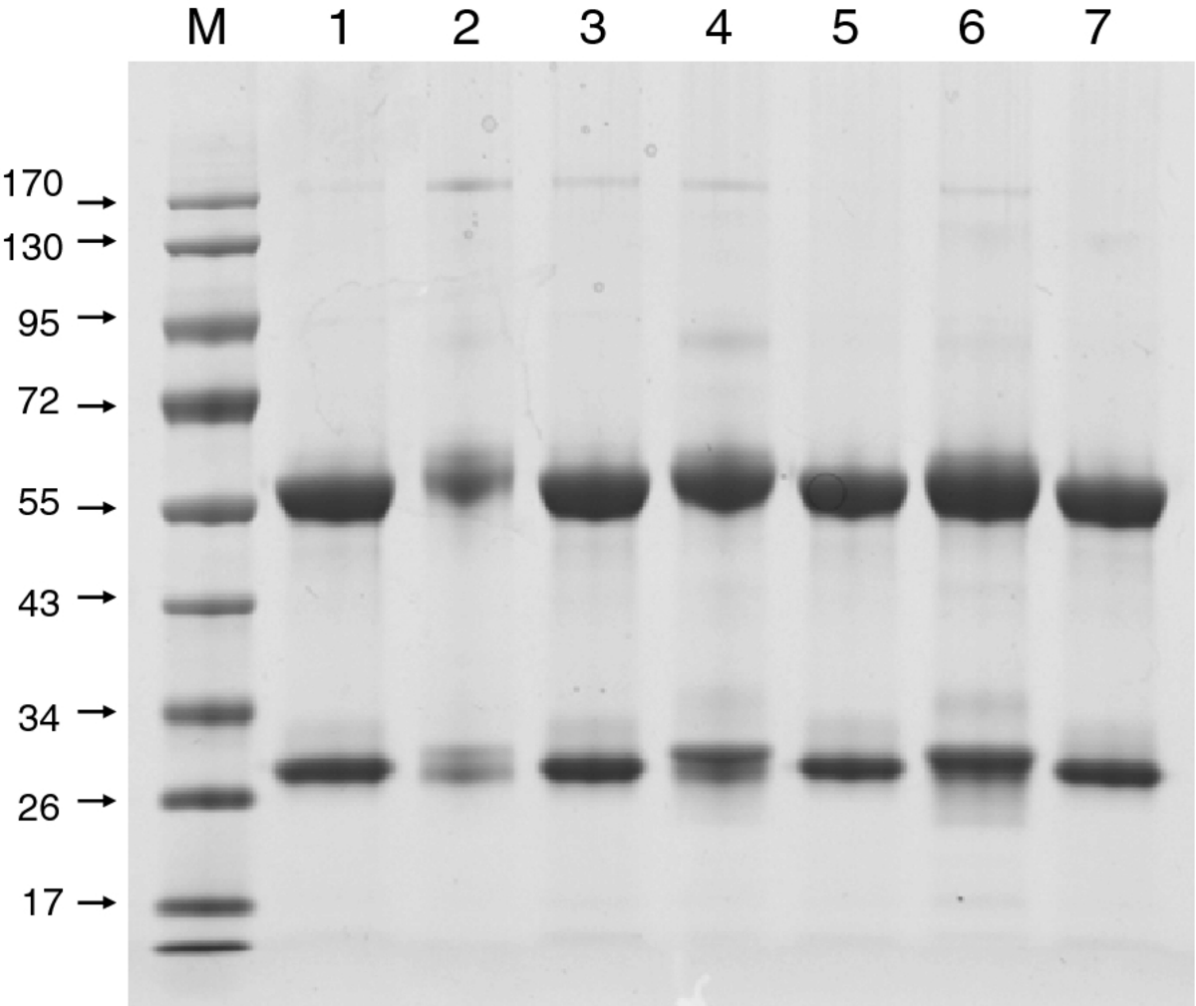
**Coomassie stain of protein A purified mAb after SDS-PAGE separation**. Lane M, size marker with molecular weight labeled on the left (in KDa); Lane 1, mAb A, 25 μg; Lane 2, mAb B, 2 μg; Lane 3, mAb C, 25 μg; Lane 4, mAb D, 10 μg; Lane 5, mAb 3C4, 25 μg; Lane 6, mAb 4H2, 25 μg; Lane 7, mAb 6B8 25 μg. Sample volumes were adjusted for their contents; mAb B and D can only attain 2 and 10 μg even at maximum volume.

All 7 mAb were purified to near homogeneousity by protein A resin (Figure 1), but the yield of mAb from those 7 clones varied significantly (Table 2). Clone A, C, 3C4, and 6B8 are high yield but clone B, D, and 4H2 are low yield (Table 2). The purified mAb was not diluted to uniform concentrations, because we would like to observe the virus neutralizing effect in the presence of highest mAb concentration possible.

**Table 2:** Monoclonal antibody yield from protein A purification of mouse ascites

Clone	Content^1 ^(μg/μL)	Volume(mL)	Yield^2 ^(mg)	Deglycerol^3 ^(μg/μL)
A	14.5	2	18.0	10.9
B	1.1	0.2	0.2	0.1
C	18.0	1.7	30.0	17.4
D	3.3	0.2	0.7	0.5
3C4	56.0	1.1	59.0	31.8
4H2	9.9	0.4	3.8	5.7
6B8	50.0	0.6	27.9	28.3

^1^Monoclonal antibody contents in selected best fractions after protein A purification and Centricon enrichment before the addition of 50% glycerol. The immunoglobulin content was determined by Bradford method with BSA as the standard.

^2^The yield was obtained by multiplying content (μg/μL) with the volume (mL).

^3^The mAb were stored at -;80°C freezer in the presence of 50% glycerol. The glycerol can interfere with the virus infection assay, so it is removed by another round of protein A purification and the immunoglobulin content was determined again

**Figure 2:**
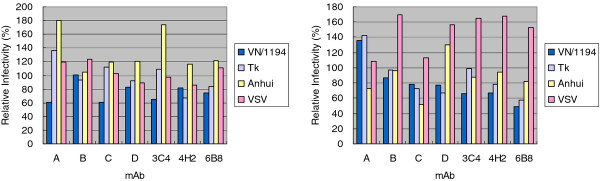
**Preliminary test of pseudovirus neutralization by mAb**. Pseudovirus, Vietnam 1194, Turkey, Anhui, and VSV at 50x TCID_50 _were premixed with 10 μL mAb for 1 hour at 37°C before infecting MDCK cells in a total volume of 100 μL. Due to disparity in Ab yield from the ascites, protein contents in purified mAb vary significantly and antibody used in each assay is 109, 1, 174, 5, 318, 57, 283 μg for clone A, B, C, D, 3C4, 4H2, 6B8, respectively (left panel). The right panel is neutralization of pseudovirus infection by pepsin treated mAb. The amount of antibody used in each assay is equivalent to 3.7 μL of mAb, which are 40.3, 0.4, 64.4, 1.9, 117.7, 21.1, 104.7 μg for clone A, B, C, D, 3C4, 4H2, 6B8, respectively, see Method for detail. In both experiments, a control reference group that received no antibody was performed in parallel for each pseudovirus and its observation was considered 100%.

**Figure 3:**
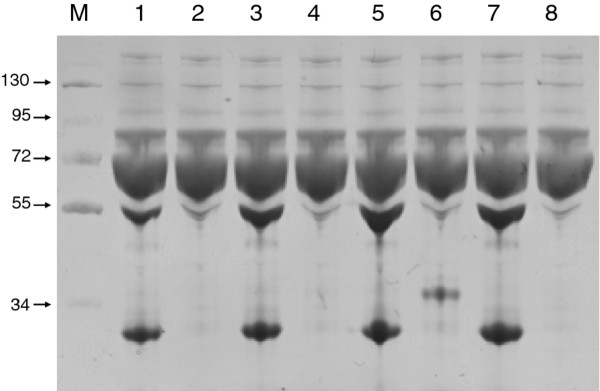
**Coomassie stain of pepsinized mAb after SDS-PAGE separation**. Lane M, size marker with molecular weight labeled on the left (in KDa); Lane 1-7 are loaded with 20 μL of pepsin digestion products as mentioned in Figure 2. Lane 1, mAb A, 16.1 μg; Lane 2, mAb B, 0.1 μg; Lane 3, mAb C, 25.8 μg; Lane 4, mAb D, 0.7 μg; Lane 5, mAb 3C4, 47.1 μg; Lane 6, mAb 4H2, 8.4 μg; Lane 7, mAb 6B8 41.9 μg; Lane 8, pepsin 7.4 μg alone.

Since H5N1 avian influenza virus is highly pathogenic and is not available for general laboratory use, we opted for a pseudovirus that expresses H5 and N1 surface proteins and contains a firefly luciferase reporter gene so that its infectivity can be assessed by luciferase activity. A preliminary neutralization scan was summarized in Figure 2 left panel. Initial screen for antibody efficacy revealed that most clones have slightly more effective suppression on Vietnam 1194 pseudovirus than others, but the overall suppressive effect is weak. Because the polybasic cleavage site is located deep near the viral envelop, away from the viral surface, epitope accessibility on the hemagglutinin may play a role in the neutralization assay [33]. Therefore, pepsin digestion on mAb was employed to cleave off the Fc domain and we examine if F (ab')_2 _can result in a better suppression during the pseudovirus infection assay. After pepsin treatment (Figure 3), the efficacy of those 7 F(ab')_2 _remained low and none of the clones provided more than 50% suppression on pseudovirus infection (Figure 2 right panel).

**Figure 4:**
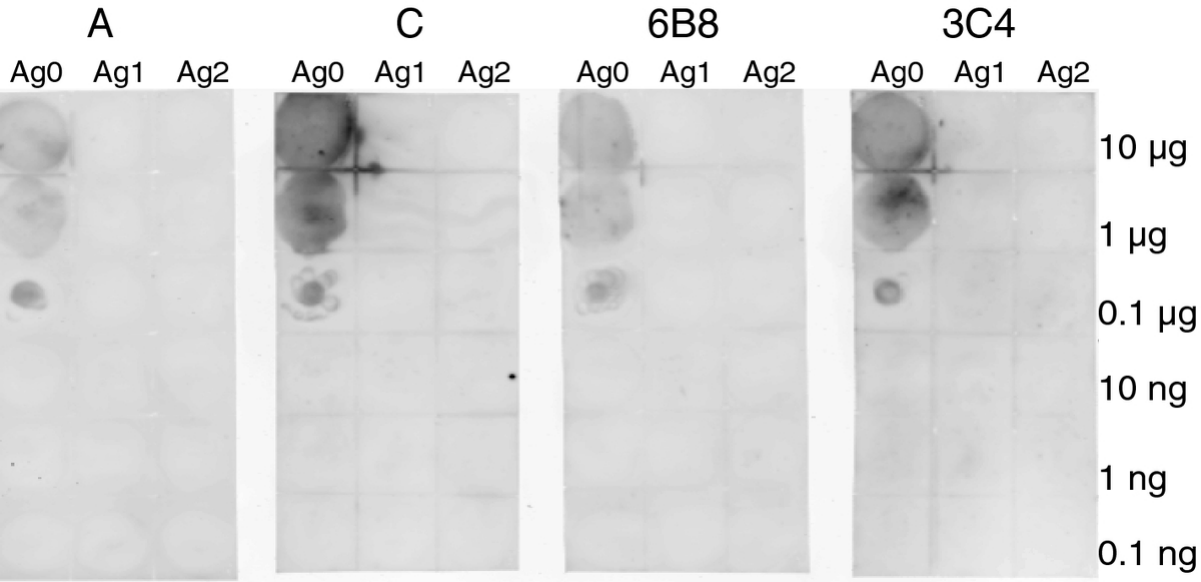
**Epitope peptide analysis by dot blot**. The full length 18 meric peptide is designated as Ag0, the N-half 8 meric peptide is designated as Ag1 and the C-half 10 meric peptide is designated as Ag2. Monoclonal antibodies A, C, 6B8 and 3C4 are immunoreactive to the full length peptide but not either half peptide. The immunoglobulin contents are 9.8, 40.0, 7.7 and 15.0 μg/μL for clone A, C, 6B8 and 3C4, respectively, and were diluted 1:500 with 2% non-fat milk in PBS. Clone B, D, and 4H2 are not immunoreactive to any of these 3 peptides at up to 10 μg, data not shown.

**Figure 5:**
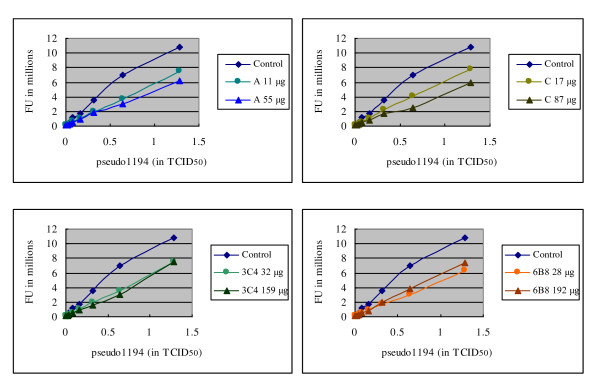
**Suppression of MDCK cell infection by pseudovirus 1194 with mAb A (top left), C (top right), 3C4 (lower left), and 6B8 (lower right)**. Y-axis indicates transfected firefly luciferase activity, expressed as fluorescence unit (FU). Pseudoviruses were premixed with mAb for 4 hours prior to infection on MDCK cells. All mAb showed substantial but weak suppression of infection by pseudovirus 1194. A concurrent series of virus dilutions were performed in parallel in the absence of mAb as the control.

All 7 monoclonal antibodies were subjected to epitope peptide analysis by dot blot. The result showed that clone A, C, 3C4 and 6B8 recognize the full length 18 meric peptide only and are reactive to as little as 0.1 μg of full length peptide (Figure 4, third row from the top). Other three clones, B, D, 4H2 are not immunoreactive to any of those peptides (up to 10 μg, data not shown). Since mAb A, C, 3C4, and 6B8 are reactive to the full length epitopic peptide (Figure 4), they were chosen for a more detailed titration, as shown in Figure 5. All 4 mAb showed suppressive effect when premixed with pseudovirus 1194 but the magnitude of suppression is again relatively small (approximately 45%).

**Figure 6:**
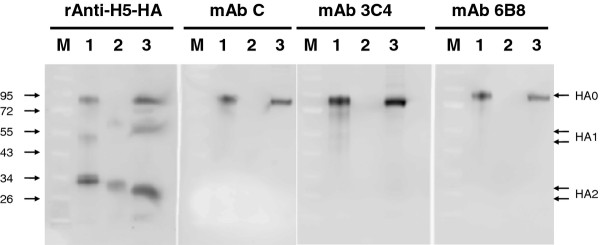
**Immunostaining of recombinant H5-HA (lane 1, 85 kDa), H5N1 vaccine virus lysate (lane 2) and pseudovirus 1194 lysate (lane 3) by polyclonal anti-HA5 Ab, mAb C, 3C4, or 6B8**. M indicates lanes for protein size markers. The anti-HA polyclonal antibody recognizes HA0 or HA2 (25-35 kDa) better than HA1 (50-60 kDa; left panel) but mAb C, 3C4, and 6B8 recognize intact hemagglutinin HA0 only (right 3 panels). The H5N1 vaccine virus has the polybasic site removed, so it is not recognized by mAb C, 3C4, or 6B8 (lane 2 in each of right 3 panels). Based on densitometry analysis, it is estimated that the ratio of HA0 (full length) to HA1+HA2 (proteolytic products) are about 1:3 on recombinant HA or pseudovirus 1194 lysate (left panel, lane 1 and 3).

To verify if these purified mAb can bind to hemagglutinin, mAb C, 3C4, and 6B8 were subjected to immunoblotting against recombinant hemagglutinin or virus lysate, as shown in Figure 6. The immunoblot indicated mAb C, 3C4, and 6B8 recognized the intact hemagglutinin (HA0) but not its cleaved products, HA1 or HA2 (Figure 6, right 3 panels) which is in agreement with the result of epitopic peptide dot blotting (Figure 4). With the use of polyclonal antibody against H5, analysis by densitometry revealed that 3 quarters of hemagglutinin has already been cut regardless whether it is viral origin or recombinant hemagglutinin (Figure 6, left panel). When native H5 hemagglutinin expressed by human embryonic kidney cells were subject to mAb A, C, 3C4, 6B8 immunostaining, the stain intensities on 293 cells were rather weak (data not shown). The weak staining of native hemagglutinin is in agreement with the fact that the majority of HA on the influenza virus are cleaved before the virus is released from its infected host.

## Conclusions

Because our mAb cannot recognize those cleaved HA1 or HA2 (Figures 4 and 6), the state of hemagglutinin cleavage on the pseudovirus would explain why our mAb have limited influence on virus infection in Figures 2 and 5. The small magnitude of suppression on pseudovirus infection (no more than 50%) can be explained by the fact that the majority of hemagglutinin on the pseudovirus is already cut and too late for our mAb to exert any suppressive effect. In order to reach the full potential of those mAb that target HA cleavage site, one may need an inhibition on those membrane bound proteases, like furin, proprotein convertase 5/6, or type II transmembrane serine proteases, like TMPRSS2, 4, HAT [8,9], so that these mAb can recognize its intact uncut epitope and prevent subsequent viral infection.

## Competing interests

The authors declare that they have no competing interests.

## Authors' contributions

HJT is responsible for the overall research; LAC carried out the epitope peptide analysis and 293 cell expressed HA immunostain study; ALY supervised the overall progress. All authors have read and approved the final manuscript.
